# Early Diagnosis of Prostate Cancer from the Perspective of Chinese Physicians

**DOI:** 10.7150/jca.36697

**Published:** 2020-03-05

**Authors:** Wei Yu, Liqun Zhou

**Affiliations:** Department of Urology, Peking University First Hospital, Institute of Urology, Peking University, National Urological Cancer Center of China, Beijing, China

**Keywords:** prostate cancer, biomarkers, risk predictive models

## Abstract

Prostate cancer (PCa) is the seventh most diagnosed cancer and the tenth leading cause of cancer mortality in China. Unlike the USA, both incidence and mortality continue to increase. In China, PCa is often diagnosed at a locally advanced or metastatic stage, resulting in a high mortality-to-incidence ratio. Implementing regular screening using a well-validated biomarker may result in the earlier diagnosis of localized disease. Furthermore, it is important to be able to distinguish between low-grade and high-grade disease, to avoid subjecting patients to unnecessary biopsies, undertreatment of significant disease, or overtreatment of indolent disease. While prostate-specific antigen (PSA) is commonly used in PCa screening around the world, its relationship to PCa is still unclear and results vary widely across different studies. New biomarkers, imaging techniques and risk predictive models have been developed in recent years to improve upon the accurate detection of high-grade PCa. Blood- and urine-based biomarkers, such as PSA isoforms, prostate cancer antigen 3, or mRNA transcripts, have been used to improve the detection of high-grade PCa. These markers have also been used to create risk predictive models, which can further improve PCa detection. Furthermore, multiparametric magnetic resonance imaging is becoming increasingly accessible for the detection of PCa. Because of ethnic variations, biomarkers and risk predictive models validated in Western populations cannot be directly applied to Chinese men. Validation of new biomarkers and risk predictive models in the Chinese population may improve PCa screening and reduce mortality of this disease in China.

## Introduction

In 2018, prostate cancer (PCa) was estimated to have the second highest incidence of cancers globally, contributing to 3.8% of deaths from cancer 1. In China, PCa was estimated to be the seventh most commonly diagnosed cancer and the tenth leading cause of cancer mortality in men 2,3. Unlike the US, which has seen a decrease in incidence and mortality, both incidence and mortality are increasing in China.^2^,^4^ The difference in mortality trends may be attributed to the establishment of regular PCa screening in the US, resulting in most incidences of PCa being diagnosed when the disease is localized 4. In China, PCa is often diagnosed at a locally advanced or metastatic stage, which may contribute to the high mortality-to-incidence ratio, close to 50% 2. This may be due to the lack of mandatory or regular screening in China, as well as a lack of well-established biomarkers or screening methods in the Chinese population. Implementation of regular screening may result in earlier diagnosis of localized disease.

Prostate-specific antigen (PSA) is a marker related to the early diagnosis of PCa and is used for PCa screening around the world. While positive biopsy results correlate with elevated serum levels of PSA, the relationship between PSA and PCa varies greatly between different studies 5,6. Furthermore, the use of total PSA levels as a diagnostic decision-making tool for biopsies has a high rate of both false-negative and false-positive results, leading to delayed diagnoses as well as unnecessary biopsies. Thus, using PSA alone can lead to over-diagnosis 7. Multiparametric magnetic resonance imaging (mpMRI) may also be used prior to transrectal ultrasound (TRUS)-guided biopsy. However, while mpMRI has a high sensitivity for PCa, it is less specific than both biopsy and PSA 8. As such, the usefulness of mpMRI alone in the early diagnosis of PCa is limited.

This situation emphasizes the need for effective biomarkers and imaging techniques which, when combined with clinical factors, can help establish effective risk predictive models to improve earlier diagnosis of PCa. This review provides an update on recent developments in PCa diagnostics, screening tools and risk predictive models, and their applicability to the Chinese population.

## The value of new biomarkers in the early diagnosis of prostate cancer

TRUS-guided biopsies are required for the diagnosis of PCa, but carry a risk of adverse events, including sepsis 9. Furthermore, biopsies can identify the presence of low-grade tumors that pose minimal risk of progression, resulting in overtreatment of the patient. An accurate pre-biopsy triage of patients who are suspected of having PCa would reduce the number of unnecessary biopsies, avoiding the pain and the risk of other adverse events associated with biopsies. Additionally, a minimally invasive prebiopsy test could be carried out more frequently for screening purposes, allowing earlier detection of aggressive disease.

### Prostate cancer antigen 3 (PCA3)

PCA3 is an overexpressed non-coding RNA in PCa, but undetectable in normal tissue or other tumor types 10. The PCA3 score is calculated as a ratio of PCA3 mRNA/PSA mRNA and has been demonstrated to be highly accurate in predicting PCa, more so than PSA alone 11,12. The US Food and Drug Administration (FDA) approved the use of a PCA3 test for the detection of PCa, using a PCA3 score cut-off of 25 to determine the likelihood of a positive diagnosis 13. Although the optimal cut-off score is still subject to debate, an increasing PCA3 score strongly correlates with a higher probability of PCa overall, as well as high-grade PCa 12,14. In a Chinese study, a decision curve analysis indicated that a PCA3-based model had a superior net benefit at almost all threshold probabilities compared with percentage-free PSA (%fPSA) or PSA density (PSAD)^15^.

### Prostate Health Index (PHI)

The PHI is a risk score calculated using a combination of PSA isoforms, [-2]proPSA (p2PSA), %fPSA and total PSA (tPSA), each of which improves prediction of PCa compared with PSA alone.^16^ PHI was found to be significantly more accurate in predicting the presence of PCa in men with negative digital rectal examinations (DRE) and a tPSA of 2-10 ng/mL than with tPSA alone, which could not differentiate between individuals with or without PCa.^17^ More importantly, while PCA3 was more accurate at predicting PCa overall, PHI was the most accurate predictor of significant PCa when compared with PCA3, tPSA, or PSAD, and correlated well with the Gleason Score 16,18,19. This was true in both European and Chinese populations 18,20. The addition of age, DRE and/or prostate volume (PV) to the PHI did not increase its predictive power in identifying high-grade PCa in Chinese men.^20^ PHI may increase the ability to detect PCa while decreasing the number of unnecessary biopsies by almost 50%; only a few cases of clinically significant PCa are likely to be missed using this index 19. PSA can sometimes be elevated above 10 ng/mL for benign tissue, which can cause a false-positive result; however, the PHI was demonstrated to accurately identify the presence of high-grade PCa even in men with significantly elevated serum PSA 19. The PHI has been approved by the FDA for the detection of PCa in men aged 50 years and older and a tPSA level of 4-10 ng/mL and negative DRE 21.

### 4Kscore®

4Kscore® (OPKO Health, Miami, USA) is a commercially available PCa testing kit, using a combination of tPSA, %fPSA, intact PSA and human Kallikrein 2 (hK2) with age, DRE results and history of prior biopsies 22. This test is able to distinguish between the presence and absence of high-grade PCa, as well as identifying patients with tumors that are likely to progress. The test also increases the predictive discrimination for 15-20-year risk of PCa mortality among men with elevated PSA 22,23. The 4Kscore® can be used to reduce the number of unnecessary biopsies by over 30% in men with elevated PSA, with little risk of missing high-grade disease 24,25. As of December 2018, the 4Kscore® test was not FDA-approved, but is offered as a Laboratory Developed Test through certified and accredited laboratories and wholly owned subsidiaries of OPKO Health.

### TMPRSS2: ERG

In addition to serum biomarkers, urinary biomarkers are also of increasing interest. Fusion of the *TMPRSS2* (transmembrane protease, serine 2) and *ERG* (a member of the E26 transformation-specific family of oncogenes) genes is highly specific for PCa, as well as displaying 93.2% specificity for predicting clinically significant PCa.^12^,^26^ This gene fusion has been found in approximately 50% of PCa patients, and fused RNA transcripts can be detected in the urine, providing the added benefit of being a non-invasive test 27. *TMPRSS2*:*ERG* mRNA is elevated in men with PCa, and is associated with tumors with a high Gleason Score 28. *TMPRSS2*:*ERG* also correlated with tumor dimensions at the time of prostatectomy, and this urine test had a greater area under the receiver operating characteristic (ROC) curve (AUC) than serum PSA for the detection of PCa 28. However, *ERG* nuclear expression (which is correlated to *TMPRSS2*:*ERG* rearrangement) is significantly lower in PCa samples for men of Chinese origin than samples from the UK, suggesting that *TMPRSS2*:*ERG* rearrangement may not be a suitable biomarker for detecting PCa in Chinese men.^29^ Currently, the use of this biomarker has not been approved by the FDA.

### SelectMDx® and ConfirmMDx®

SelectMDx (MDxHealth, Irvine, USA) is a commercially available, urine-based test for PCa. *DLX1* and *HOX6* expression is associated with an increased likelihood of high-grade PCa, and mRNA transcripts of these two genes can be detected in urine 30. The combination of these two mRNA markers with traditional clinical parameters, including PSA, PSAD, DRE, age and family history, is used in the SelectMDx test 31. Another test developed by MDxHealth is ConfirmMDx, which uses residual cancer-negative prostate biopsy samples. The basis of ConfirmMDx is the 'field effect', whereby molecular changes, such as epigenetic changes, occur in benign-looking tissue adjacent to cancerous cells 32. These changes cannot be detected by histopathology, but can be detected using methylation-specific polymerase chain reaction (MSP). Abnormal methylation of genes such as *GSTP1, APC* and *RASSF1* is often detected in PCa. ConfirmMDx uses multiplex MSP to determine the epigenetic status of these genes in histopathologically negative tissue, and aids the detection of occult PCa 33. To date, neither SelectMDx nor ConfirmMDx has obtained FDA approval.

While the biomarkers outlined above and screening techniques are improving the accuracy of early diagnosis, each technique has its limitations, calling for the need to develop better diagnostic methods/models to improve the early detection of PCa.

## The value of mpMRI in the early diagnosis of prostate cancer

mpMRI may also be widely used as a triage test prior to TRUS-guided biopsy. The PROMIS study evaluated mpMRI in men with elevated PSA levels, followed by TRUS-guided biopsy and template prostate mapping (TPM) biopsy 9. Of the three techniques used, mpMRI was the most sensitive in detecting clinically significant PCa, and patients negative for mpMRI may be able to avoid unnecessary biopsies 8,9. However, mpMRI was less specific than both biopsy techniques, with a high false-positive rate; therefore, patients positive for PCa by mpMRI still require a biopsy to confirm the presence of clinically significant disease prior to treatment 8,9. Because mpMRI is a subjective test, there is a risk of interobserver variability; the effectiveness of mpMRI depends on the skill of the technician performing the analysis, and can lead to reproducibility issues 34. A MRI suspicion score (mSS), utilizing MRI-ultrasound fusion targeted prostate biopsy (MRF-TB), was used by Meng *et al.* to provide a grading of cancer suspicion and predict the likelihood of aggressive disease 35. While overall detection of PCa was not significantly different to standard TRUS-guided biopsies, MRF-TB was superior in detecting high-grade PCa, while also noticing fewer low-grade PCa in men who had not previously undergone prostate biopsies 35,36. Furthermore, a higher mSS score was associated with a higher detection rate of high-grade tumors 35. In future, lowering costs of mpMRI and other imaging techniques mean that these techniques will become more accessible. If mpMRI can be validated in different populations, and as it becomes widely available, it will be an invaluable tool in the early detection of significant tumors, helping to limit unnecessary biopsies in men with no or indolent disease.

Imaging techniques, such as mpMRI, are improving the accuracy of early diagnosis, but each individual technique has its limitations. Risk predictive models combining multiple variables to determine the possibility of PCa may be able to further improve the early diagnosis of PCa.

## The value of risk predictive models in the early diagnosis of prostate cancer

Given that using individual biomarkers may not always be accurate, combining multiple variables has been trialled to reduce the limitations of each technique and increase predictive ability. The variables used in these risk calculators are detailed in Table 1.

Two particularly prominent risk calculators have been developed from large PCa screening trials in the US and Europe: the Prostate Cancer Prevention Trial Risk Calculator (PCPT-RC) and the European Randomized Study of Screening for Prostate Cancer Risk Calculator (ERSPC-RC).

### Prostate Cancer Prevention Trial Risk Calculator (PCPT-RC)

The PCPT-RC was developed with a cohort of 5,519 men over the age of 55 years with a PSA value less than 3.0 ng/mL. This risk calculator is available for use online and takes into account race, age, DRE, PSA, family history and prior biopsy results 37. While the PCPT-RC score correlated with the risk of PCa, the ROC curve for the risk calculator was not statistically significantly different to PSA alone, with an exception for African-American men, where the PCPT-RC outperformed PSA alone 38. In 2014 PCPT-RC 2.0 was released, which included the %fPSA into the calculator. Although this improved the identification of significant disease, it was not able to distinguish between high- and low-grade diseases, or low-grade disease and absence of disease 39.

### European Randomized Study of Screening for Prostate Cancer Risk Calculator (ERSPC-RC)

ERSPC-RC is a series of multistep risk calculators that can be used at various stages of screening and does not require blood tests 40. ERSPC developed six risk calculators, which were dependent on the information available, such as PSA, PHI or Gleason Score. The newest of the risk calculators, ERSPC-RC6, can be used to determine the likelihood of PCa for the proceeding four years 40. After 13 years of follow-up, the ERSPC-RC was able to reduce unnecessary biopsies by reducing the number of screenings needed to prevent one PCa death, from 1,055 to 781 men 41. The ERSPC-RC is significantly better at predicting PCa overall and specifically for high-grade PCa than the PCPT-RC (AUC: 0.741 [95% CI, 0.717-0.763] vs 0.692 [95% CI, 0.668-0.716], *P* < 0.001) 42. However, the ERSPC-RC tended to under-predict the presence of PCa, resulting in men with PCa who were left untreated 42.

The ability to detect the presence of PCa was not significantly different between ERSPC-RC and PCPT-RC 2.0; however, in detecting significant PCa, the AUC for the ERSPC-RC was significantly higher than for the PCPT-RC 2.0 (0.73 vs 0.70; DeLong test, *P* = 0.043) 43. Decision curve analyses indicated that both risk calculators provide a clinical net benefit in the threshold probability range, between 18% and 40% for any PCa and between 8% and 40% for clinically significant PCa 42,43.

### Predictive models combining new biomarkers can improve early diagnosis of prostate cancer

To increase the net benefit from these risk calculators, other biomarkers have been included with these risk predictive models. Loeb *et al.* investigated the value of adding PHI to the PCPT-RC or ERSPC-RC in a population of 728 men with PSA levels of 2-10 ng/mL and negative DRE 43. Discrimination of aggressive disease was significantly improved by the addition of PHI to both risk calculators, and a new model was designed including age, previous biopsy, PV, PSA and PHI for the prediction of significant PCa. This new model offered further improvement to the PCPT-RC or ERSPC-RC, and showed a net benefit for the model at threshold values greater than 3% 44. Similarly, the addition of PCA3 or *TMPRSS*:*ERG* to PCPT-RC or ERSPC-RC also improved the predictive value of these risk calculators 12,14,28.

The Stockholm 3 (STHLM3) study, established in Sweden, utilized a combination of plasma protein biomarkers (PSA, fPSA, intact PSA, hK2, microseminoprotein-beta [MSMB] and macrophage inhibitory cytokine 1 [MIC1]), genetic markers (based on single nucleotide morphisms of 254 genes, including *HOXB13*), clinical variables (age, family history and previous biopsy) and prostate exam results (DRE and PV) to develop a risk predictive model 45,46. When STHLM3 was compared with the PSA test alone, at the same sensitivity, it significantly improved the specificity of PCa detection and was able to reduce the number of unnecessary biopsies by 44% 45,47.

The Mi-Prostate score (MiPS) combines PCA3 and TMPRSS:ERG scores with PSA levels to ascertain the risk of PCa 48. Addition of the TMPRSS:ERG score to PCA3 and total PSA significantly improved the AUC compared with PCA3 plus PSA or TMPRSS:ERG plus PSA 48. Addition of MiPS to the PCPT-RC increased the net benefit of the model and further reduced unnecessary biopsies across all threshold probabilities for PCa, and specifically high-grade PCa 48. However, this is the only study using the MiPS score with the PCPT-RC, and will require further validation in more studies and patient populations before widespread use as a risk predictive model for PCa.

### Including mpMRI in predictive models can improve the early diagnosis of prostate cancer

In addition to the incorporation of new biomarkers into risk predictive models, mpMRI has also been used to enhance the predictive power of these models. The addition of the Prostate Imaging Reporting and Data System (PI-RADS) score, an evaluation score based on mpMRI, to a risk calculator with age, PSA, and DRE-PV significantly increased the AUC, compared with risk calculators without mpMRI 49,50. Inclusion of mpMRI into the risk calculation also reduced the number of unnecessary biopsies, while missing few high-grade PCa. A risk model developed by Radtke *et al.* incorporated PSA, PV, DRE, age, and the PI-RADS score to determine the risk of significant PCa 51. Two separate nomograms were developed for men who were biopsy-naïve and those who had a previous biopsy. The risk model was comparable to that of ERSPC-RC3 (for biopsy-naïve men) with PI-RADS, while in men with a previous biopsy the discrimination of this risk model was better than that of ERSPC-RC4 (for men with previous biopsies) with or without a PI-RADS score. Both risk models used by Radtke *et al.* produced a higher net benefit compared with the ERSPC-RCs in terms of detecting patients with significant PCa, and may provide a clinically useful tool for men in whom biopsy is being considered 51. van Leeuwen *et al.* further incorporated previous biopsy into a risk model with the same parameters used by Radtke *et al.* 52. The inclusion of the PI-RADS score into the risk model significantly improved prediction of significant PCa compared to risk models excluding PI-RADS 52.

A recent study investigated the use of the PI-RADS version 2 score with age, PSA, PSAD and PV to calculate the prostate biopsy rating scale (PBRS) in Chinese men 53. The PBRS score was found to reduce the number of unnecessary biopsies by up to 63%, when compared to using PSA alone, which is the current standard of PCa detection in China. 53 The PBRS also demonstrated improvements when compared to the biomarkers alone in accurately in the AUC curve for all PCa, PCa with a Gleason score ≥ 7, PCa with a clinical stage ≥ T2b, and PCa with D'Amico risk > low. However, this was a retrospective analysis and many men with PSA levels of 4-10 ng/ml refused biopsies and opted for follow-up observation and were underrepresented in this study. As such, while the PBRS shows promise, further investigation is required to validate this model 53.

### Predictive models that combine new biomarkers and mpMRI may improve early diagnosis of prostate cancer

New biomarkers and mpMRI have improved the accuracy of PCa risk prediction compared with more traditional parameters, such as tPSA and DRE. The use of new biomarkers and mpMRI together have the potential to further improve risk prediction. The combination of mpMRI and PCA3 improved the accuracy of predicting prostate biopsy outcomes when compared with PCA3 alone (0.726 vs 0.750) 54. Furthermore, the addition of mpMRI to PCA3 testing also improved sensitivity (0.793 vs 0.680), positive and negative predictive values and the AUC (0.857 vs 0.825; *P* < 0.001) 54. When mpMRI or a new biomarker (PHI and PCA3) was added to a base model of DRE and age, mpMRI improved risk prediction, but not with biomarkers alone 55. In this study, the further addition of PHI or PCA3 to the mpMRI model did not provide further benefit in ROC or decision curve analyses 55. This was corroborated by Gnanapragasam *et al*., who also found that the addition of PSA or PHI to mpMRI results did not generate a clinically relevant benefit 56. However, the addition of PHI (at a cut-off ≥ 35) was able to significantly improve the predictive performance for detecting clinically significant cancers (negative predictive value of 0.97 and sensitivity of 0.99) 56. Furthermore, PHI was able to predict the presence of significant PCa in men with negative mpMRI, reducing the likelihood of missed PCa 56.

The inclusion of clinical factors, such as new biomarkers and/or mpMRI, to risk calculators increases the ability to distinguish high-grade and low-grade PCa, compared with risk calculators without clinical factors, or clinical factors alone. These non-invasive and minimally invasive tests can reduce the number of unnecessary biopsies and overtreatment, while still identifying patients who require aggressive therapy. The AUC for these biomarkers and risk predictive models is summarized in Table 2.

## Risk predictive models for Chinese populations

While the PCPT-RC and ERSPC-RC fit the European and American populations, they have not been tested in Chinese cohorts, and ethnic variations suggest that they may not be as reliable. To rectify this, studies have been conducted to develop PCa risk calculators that are optimized for the Chinese population. A multiplex model using urinary PCA3, TMPRSS:ERG, Annexin A3 and Sarcosine was developed by Cao *et al.*, and was found to perform significantly better than PSA alone in predicting PCa in Chinese men 57. However, this study did not distinguish between the identification of low- and high-grade disease; thus, it would not be useful in the reduction of unnecessary biopsies 57. On the other hand, a PHI-based nomogram developed by Zhu *et al.* using PHI, age and PV was effective in predicting PCa, and would reduce the number of unnecessary biopsies by 27% 58. A separate study by Chiu *et al.* also used PHI, age, and PV to develop a risk calculator in a Chinese cohort and was able to reduce more unnecessary biopsies than PSA alone, PSA, DRE-PV with age, or PHI alone at all risk thresholds; 80.2% of unnecessary biopsies could be avoided at the 20% risk threshold for high-grade PCa 20. This risk calculator was also able to reduce the number of biopsies when specifically looking for high-grade disease.

The Huashan risk calculators developed by Wu *et al*. were better able to identify Han Chinese patients with PCa than the PCPT-RC, using age, DRE result, PV, PSA, %fPSA and TRUS-guided biopsy results (Huashan RC1)^59^. In particular, the Huashan RC1 had a greater AUC compared with PCPT-RC and the Huashan RC2, and thus able to reduce the greatest number of unnecessary biopsies. Another model, the Prostate Cancer Predictor (PCP), used a combination of tPSA, fPSA, and complexed PSA (cPSA) to develop a model that was strongly correlated with PCa 49. PCP had a greater specificity for PCa than tPSA, fPSA, %fPSA, or cPSA alone and was able to reduce the number of unnecessary biopsies (22.8% vs 11.1%, 11.2%, 17.4% and 15.5%, respectively) 49. A study in Shanghai established the Chinese Prostate Cancer Consortium Risk Calculator (CPCC-RC), which incorporated age, logPSA, logPV, fPSA and DRE to calculate the risk of PCa (model 1) or high-grade PCa (model 2) 60. The CPCC-RC showed better accuracy and clinical benefit in the Chinese population than either the ERSPC-RC or PCPT-RC, with an AUC of 0.801 (95% CI, 0.771-0.831) and 0.826 (95% CI, 0.796-0.857) for models 1 and 2, respectively 60.

As the use of mpMRI becomes more common in China, PI-RADS scores can be incorporated into risk predictive models. Niu *et al.* developed a nomogram (LR model) for the detection of high-grade PCa in men with PSA levels between 4 and 10 ng/mL 61. This model, which incorporates age, adjusted-PSAD and PI-RADS v2, had an AUC of 0.85 (95% CI, 0.79-0.90), sensitivity of 87.3%, and specificity of 78.4% at a threshold risk score ≥ 0.36 61. Although no decision curve analyses were performed, the high performance of the LR model suggests that it is an effective tool in identifying high-grade PCa in Chinese men with PSA levels in the “gray zone” and, therefore, has the potential to reduce unnecessary biopsies 61.

Thus, as outlined above, many risk predictive models are well established in European and American populations. However, when these models were applied to Chinese populations, the results were less than optimal. Several models have now been developed in Chinese cohorts. Although these models still require further validation, they may better assist in the early detection of high-grade PCa in Chinese men.

## Future directions

Current risk calculators are more accurate than clinical factors (either biomarkers or mpMRI) individually, but there is still room for improvement. There are currently more than 100 ongoing studies registered on ClinicalTrials.gov investigating methods of detection or diagnosis of PCa, two of which are recruiting in China (Table 3). A further three studies involve the validation of risk predictive models and includes the use of MRI (Table 4). Other studies explore the use of biparametric MRI, positron emission tomography (PET)/computed tomography (CT) scans, including the use of radiolabeling with fludeoxyglucose (^18^F) or Gallium-68, for the detection of PCa. Eleven of the ongoing studies are currently in Phase III or IV validation (Table 5).

The subjective nature of mpMRI for diagnosis is a significant limitation to the technique, as well as the time-consuming nature of image analysis. However, this may be overcome in the future through computer-aided detection, which has been relatively successful in the analysis of mammograms in breast cancer, as well as CT colonography. Over the past decade, many studies were performed in patients suspected of PCa, but most of these had small sample sizes with fewer than 100 participants (summarized by Afef *et al.*) 62. Computer-aided detection and/or diagnosis can remove interoperator variability, provided a single, optimized algorithm is used, as different systems will have differing levels of accuracy 62. The algorithm developed by Roethke *et al.* was comparable in accuracy to human analyses, although this study used a small sample size and the older version of the PI-RADS scoring system 63.

Identification of new molecular drug targets is key to the development of effective treatments for advanced PCa 64. A recent study combining four profiles from the Gene Expression Omnibus identified epithelial cell adhesion molecule (EPCAM), twist family basic helix-loop-helix transcription factor 1 (TWIST1), CD38, and vascular endothelial growth factor A (VEGFA) as hub genes which may be potential therapeutic targets in PCa. Promising results have been demonstrated with agents targeting CD38 and VEGFA in the treatment of myeloma and renal carcinoma, respectively 64. These agents may also be applied for treatment of PCa, upon further validation of these targets and is worth investigating.

## Conclusions

Early diagnosis is essential for reducing PCa mortality, due to a lack of effective treatments for advanced disease. This may be accomplished through regular PCa screening using an optimized risk predictive model. New biomarkers are continually being identified and validated, which may be used to further improve existing risk predictive models and enhance their predictive powers. Nevertheless, while these, as well as existing biomarkers and risk calculators require further validation, they show promise in improving the early diagnosis of PCa in Chinese men. This will assist in lowering the PCa mortality rate in China.

## Figures and Tables

**Table 1:** Variables included in prostate cancer (PCa) diagnostic tests.

PCa diagnostic test	Age	Race	Family history	DRE	Prostate volume	Previous biopsy	TRUS	PSA	PCA3	fPSA	PHI	mpMRI	Other markers
PHI^16^								●		●			●^a^
4Kscore®^23^	●			●		●		●		●			●^b^
PCPT^38^	●	●	●	●		●		●					
PCPT 2.0^39^	●	●	●	●		●		●		●			
PCPT-PHI^44^	●	●	●	●		●		●		●	●		
PCPT-PCA3^14^	●	●	●	●		●		●	●				
ERSPC^c^ 40	●		●	●		●	●	●					
ERSPC-PHI^44^	●		●	●	●	●		●			●		
Loeb *et al.*^44^	●				●	●		●			●		
STHLM3 study^45^	●		●	●	●	●		●		●			●^d^
MiPS^48^								●	●				●^e^
Radtke *et al*.^51^	●			●	●			●				●	
Cao *et al.*^57^									●				●^f^
PHI-nomogram^58^	●				●						●		
Chiu *et al*.^20^	●				●						●		
Huashan RC1^59^	●			●	●		●	●^g^		●			
Huashan RC2^59^	●			●				●^g^		●			
PCP^15^								●		●			●^h^
CPCC-RC^60^	●			●	●			●		●			
Niu *et al.*^61^	●							●^i^				●	
van Leeuwen *et al*.^52^	●			●				●				●	
PBRS^53^	●				●			●^j^				●	

^a^PHI also used p2PSA in its calculations. ^b^Other markers in the 4Kscore® test include intact PSA and hK2. ^c^The ERSPC comprises multiple risk calculators using different combinations of variables. ^d^Other markers included in the STHLM3 test include intact PSA, hK2, MSMB, MIC1 and genetic markers. ^e^Urinary levels of *TMPRSS2:ERG*, Annexin A3 and Sarcosine were also included. ^f^TMPRSS2:ERG score was used in the MiPS. ^g^LogPSA was used for both Huashan risk calculators. ^h^PCP risk calculator included complexed PSA in its calculations. ^i^The nomogram developed by Niu *et al.* included adjusted PSA density. ^j^The PBRS utilised both PSA and PSA density. CPCC-RC: Chinese Prostate Cancer Consortium Risk Calculator; DRE: digital rectal examination; ERSPC: European Randomized Study of Screening for Prostate Cancer; fPSA: free PSA; hK2: human kallikrein 2; MIC1: macrophage inhibitory cytokine 1; MiPS: Mi-Prostate score; MSMB: microseminoprotein-beta; mpMRI: multiparametric magnetic resonance imaging; p2PSA: [-2]proPSA; PBRS: prostate biopsy rating scale; PCA3: prostate cancer antigen 3; PCP: Prostate Cancer Predictor; PCPT: Prostate Cancer Prevention Trial; PHI: Prostate Health Index; PSA: prostate-specific antigen;STHLM3: Stockholm 3; TRUS: transrectal ultrasound.

**Table 2:** Prevalence of prostate cancer and area under the ROC curve for biomarkers and risk predictive models

Biomarker/ Model	Reference	Diagnostic rate of PCa/high-grade PCa		AUC (95% CI)		Comments
PSA 6	Vickers 2010	Any PCa:	26-47%		N/A	Comparison of 10 ERSPC cohorts
PCA3 11	Haese 2008	Any PCa:	25% (118/463)	Any PCa:	0.658	Using the PROGENSA PCA3 assay, in a multinational European study in men with one or two previous negative biopsies
PHI 16	Seisen 2015	Any PCa:	45% (62/138)	Any PCa:	0.65	
		HG-PCa:	28% (39/138)	HG-PCa:	0.80	
4Kscore® 23	Punnen 2015	Any PCa:	26% (192/740)	Any PCa:	0.80-0.90	Meta-analysis of 10 studies, totaling 15,139 subjects
		HG-PCa:	5% (40/740)			
PCPT 38	Parekh 2006	Any PCa:	33% (148/446)	Any PCa:	0.655 (0.602-0.708)	
		HG-PCa:	9% (40/446)	African Americans:	0.800 (0.678-0.922)	
PCPT 2.0 39	Ankerst 2014	Any PCa:	14% (942/5468)	HG-PCa:	0.744* (0.621-0.881)	
		HG-PCa:	4% (254/54668)			
PCPT-PHI 44	Loeb 2017	Any PCa:	84% (610/728)	Any PCa:	0.696	
		HG-PCa:	16% (118/728)	HG-PCa:	0.697	
PCPT-PCA3 14	Wei 2014	Any PCa:	47% (264/562)	Any PCa:	0.79	AUCs listed for initial biopsies
		HG-PCa:	NS	HG-PCa:	0.78	
ERSPC^42^	Foley 2016	Any PCa:	58% (1,153/2,001)	Any PCa:	0.710 (0.688-0.733)	
		HG-PCa:	35% (699/2,001)	HG-PCa:	0.741 (0.717-0.763)	
ERSPC-PHI 42	Foley 2016	Any PCa:	58% (1,153/2,001)	Any PCa:	0.757 (0.692-0.822)	
		HG-PCa:	35% (699/2,001)	HG-PCa:	0.778 (0.708-0.847)	
STHLM3 study 45	Grönberg 2015	Any PCa:	37% (2,295/6,221)	Any PCa:	0.69 (0.68-0.71)	Men aged 50-69 years from Stockholm, Sweden, randomly selected from the Swedish Population Register, excluding men with prevalent PCa at recruitment
		HG-PCa:	15% (921/6,221)	HG-PCa:	0.74 (0.72-0.75)	
MiPS 48	Tomlins 2016	Any PCa:	42% (518/1,225)	Any PCa:	0.751	
				HG-PCa:	0.772	
Radtke *et al.* 51	Radtke 2017	HG-PCa:	42% (489/1,159)	Biopsy naïve:	0.83	Focused on identifying high-grade disease
				Previous biopsy:	0.81	
Cao et al. 57	Cao 2011	Any PCa:	65% (86/131)	PSA 4-10 ng/mL:	0.84 (0.766-0.915)	Study in a Chinese population
				All PSA:	0.856 (0.789-0.923)	
PHI-nomogram 58	Zhu 2015	Any PCa:	13% (73/577)	Any PCa:	0.786 (0.678-0.894)	Study in a Chinese population
		HG-PCa:	6.8% (39/577)			
Chiu *et al.* 20	Chiu 2016	Any PCa:	10% (62/569)	Any PCa:	0.78 (0.72-0.85)	Study in a Chinese population
		HG-PCa:	2.8% (16/569)	HG-PCa:	0.83 (0.71-0.96)	
Huashan RC1 59	Wu 2016	Any PCa:	45% (480/1059)	Any PCa:	0.849 (0.815-0.882)	Study in a Chinese population
		HG-PCa:	17% (184/1059)	HG-PCa:	0.855 (0.809-0.900)	
Huashan RC2 59	Wu 2016	Any PCa:	45% (480/1059)	Any PCa:	0.794 (0.754-0.883)	Study in a Chinese population
		HG-PCa:	17% (184/1059)	HG-PCa:	0.886 (0.842-0.929)	
PCP 15	Wang F, 2017	PSA 4-10 ng/mL:	24% (42/173)	PSA 4-10 ng/mL:	0.788 (0.701-0.876)	Study in a Chinese population
		PSA >10 ng/mL:	49% (123/250)	PSA > 10 ng/mL:	0.821 (0.761-0.880)	
CPCC-RC 60	Chen 2016	Any PCa:	34% (632/1,835)	Any PCa:	0.801 (0.771-0.831)	Study in a Chinese population
		HG-PCa:	24% (442/1,835)	HG-PCa:	0.826 (0.796-0.857)	
Niu *et al*. 61	Niu 2017	HG-PCa:	22% (50/225)	HG-PCa:	0.85 (0.79-0.90)	Focused on identifying high-grade disease; Chinese population
van Leeuwen *et al.* 52	van Leeuwen 2017	HG-PCa:	38% (149/393)	HG-PCa:	0.883 (0.849-0.916)	Focused on identifying high-grade disease

*Median AUC (range). AUC**:** area under the receiver operating characteristic curve; CI: confidence interval; CPCC-RC: Chinese Prostate Cancer Consortium Risk Calculator; ERSPC: European Randomized Study of Screening for Prostate Cancer; HG-PCa: high-grade prostate cancer; MiPS: Mi-Prostate score; PCA3: prostate cancer antigen 3; PCP: Prostate Cancer Predictor; PCPT: Prostate Cancer Prevention Trial; PHI: Prostate Health Index; PSA: prostate-specific antigen;STHLM3: Stockholm 3.

**Table 3:** Ongoing studies currently recruiting in China investigating the diagnosis or detection of prostate cancer.

Study title	NCT number	Intervention	Geographic location	Number enrolled	Study start
Establishment and clinical assessment of a prostate cancer risk model based on the updated circulating tumor cell detection technique	NCT02940977	Other: blood draws	Shanghai, China	120	October 2016
ICG-based fluorescence imaging in localization of prostate cancer and metastatic lymph nodes	NCT02840617	Drug: ICG	Guangdong, China	50	March 2016

ICG: indocyanine green.

**Table 4:** Current ongoing studies involving risk predictive models.

Study title	NCT number	Biomarkers and/or mpMRI	Geographic location	Number enrolled	Study start
Early and accurate detection of prostate cancer in general practice	NCT03431753	STHLM3 + mpMRI PSA + mpMRI	Denmark Sweden	3,000	February 2018
MRI and biomarkers in prostate cancer (Multi-IMPROD)	NCT02241122	MRI Serum biomarkers Urine biomarkers	Finland	400	September 2014
Improved prostate cancer diagnosis - combination of rapid prebiopsy MRI and biomarkers (IMPROD2_0)	NCT02844829	MRI Serum biomarkers Urine biomarkers	Finland	200	July 2016

mpMRI: multiparametric magnetic resonance imaging; MRI: magnetic resonance imaging; PSA: prostate-specific antigen; STHLM3: Stockholm 3.

**Table 5:** Current ongoing Phase III and IV studies investigating the diagnosis or detection of prostate cancer

Study title	NCT number	Intervention	Geographic location	Number enrolled	Study start
PET/MRI in patients with suspected prostate cancer	NCT02659527	Drug: ^68^Ga-PSMA-HBED-CC PET Device: Biograph mMR, Siemens	Austria	220	January 2016
^68^Ga-PSMA PET/MRI in finding tumors in patients with intermediate- or high-risk prostate cancer undergoing surgery	NCT02678351	Drug: ^68^Ga-PSMA Procedure: MRI Procedure: PET	USA	200	June 2016
^68^Ga-PSMA in preprostatectomy patients	NCT03388346	Drug: Ga-68 PSMA-HBED-CC PET	USA	40	February 2018
^18^F-NaF PET imaging for bone scintigraphy	NCT01930812	Procedure: NaF PET/CT imaging Procedure: ^99m^Tc-medronate whole body bone scan with SPECT Drug: ^18^F-sodium fluoride	Canada	286	April 2014
^68^Ga-PSMA PET for patients with biochemical recurrence of prostate cancer	NCT03389451	Drug: ^68^Ga-PSMA-HBED-CC PET	USA	40	February 2018
Gallium-68 PSMA-11 PET imaging in patients with biochemical recurrence	NCT03353740	Drug: ^68^Ga-PSMA-HBED-CC PET	USA	500	October 2017
MRI versus PSA in prostate cancer screening	NCT02799303	Device: mpMRI Other: PSA testing	Canada	1010	June 2016
Staging prostate cancer with hybrid C11-choline PET/MR and mpMRI	NCT03404648	Drug: ^11^C choline PET tracer Drug: Gadobutrol Device: PET/MR scanner	USA	20	November 2017
^68^Ga-RM2 PET/MRI in biochemically recurrent prostate cancer	NCT02624518	Drug: ^68^Ga-labeled GRPR Antagonist BAY86-7548 Procedure: MRI Procedure: PET	USA	100	November 2015
Gallium-68 PSMA-11 PET in intermediate- to high-risk preprostatectomy patients	NCT02919111	Drug: ^68^Ga-PSMA-HBED-CC PET	USA	150	October 2016
Study of ^18^F-DCFPyL PET/CT imaging in patients with prostate cancer	NCT02981368	Drug: ^18^F-DCFPyL injection	USA	377	November 2016

CT: computed tomography; ^18^F-DCFPyL: 2-(3-(1-carboxy-5-[(6-[^18^F]fluoro-pyridine-3-carbonyl)-amino]-pentyl)-ureido)-pentanedioic acid; ^68^Ga-PSMA-HBED-CC: Gallium N,N-bis[2-hydroxy-5-(carboxyethyl)benzyl]ethylenediamine-N,N-diacetic acid; GRPR: gastrin-releasing peptide receptor; ^68^Ga-RM2^: 68^Ga-DOTA-4-amino-1-carboxymethyl-piperidine-D-Phe-Gln-Trp-Ala-Val-Gly-His-Sta-Leu-NH_2_; mMR: molecular magnetic resonance imaging; mpMRI: multiparametric magnetic resonance imaging; MRI: magnetic resonance imaging; PET: positron emission tomography; PSA: prostate-specific antigen; PSMA**:** prostate-specific membrane antigen; SPECT: single-photon emission computed tomography.

## Abbreviations

AUC: area under the curve
CPCC-RC: Chinese Prostate Cancer Consortium Risk Calculator
cPSA: complexed prostate-specific antigen
CT: computed tomography
DRE: digital rectal examination
ERSPC-RC: European Randomized Study of Screening for Prostate Cancer Risk Calculator
FDA: Food and Drug Administration
fPSA: free prostate-specific antigen
hK2: human Kallikrein 2
MIC1: macrophage inhibitory cytokine 1
MiPS: Mi-Prostate score
mpMRI: multiparametric magnetic resonance imaging
MRF-TB: magnetic resonance imaging-ultrasound fusion targeted prostate biopsy
MSMB: microseminoprotein-beta
MSP: methylation- specific polymerase chain reaction
mSS: magnetic resonance imaging suspicion score
p2PSA: [-2]pro prostate-specific antigen
PCa: prostate cancer
PCA3: prostate cancer antigen 3
PCP: Prostate Cancer Predictor
PCPT-RC: Prostate Cancer Prevention Trial Risk Calculator
PET: positron emission tomography
PHI: Prostate Health Index
PI-RADS: Prostate Imaging Reporting and Data System
PSA: prostate-specific antigen
PSAD: prostate-specific antigen density
PV: prostate volume
ROC: receiver operating characteristic
STHLM3: Stockholm 3 study
TPM: template prostate mapping
tPSA: total prostate-specific antigen
TRUS: transrectal ultrasound
